# The probiotic *Lacticaseibacillus rhamnosus* SD11 alleviates the progression of liver and colon damage through modulation of inflammation and tight junction proteins in streptozotocin-induced diabetic mice

**DOI:** 10.1371/journal.pone.0313395

**Published:** 2024-11-21

**Authors:** Waraporn Ruathong, Pissared Khuituan, Saranya Peerakietkhajorn, Rawee Teanpaisan, Jongdee Nopparat

**Affiliations:** 1 Faculty of Science, Division of Health and Applied Sciences, Prince of Songkla University, Songkhla, Thailand; 2 Faculty of Science, Division of Biological Science, Prince of Songkla University, Songkhla, Thailand; 3 Faculty of Dentistry, Research Center of Excellence for Oral Health, Prince of Songkla University, Hat Yai, Thailand; 4 Center of Excellence for Trace Analysis and Biosensor, Prince of Songkla University, Hat Yai, Songkhla, Thailand; South China Agricultural University, CHINA

## Abstract

*Lacticaseibacillus rhamnosus* SD11 (SD11) has several health benefits for the host, including antidiabetic, anti-inflammatory, and antimicrobial effects. However, the antidiabetic mechanism of SD11 has not been clearly elucidated. The current study assessed the effects of SD11 and the associated underlying mechanisms on streptozotocin (STZ)-induced diabetic mice. Compared with the normal control, SD11 supplementation for 4 weeks significantly improved the metabolic profiles, including body weight (BW), fasting blood glucose (FBG), fasting insulin level (FIN), and liver index (LI), in conjunction with a lower NAS score. A notable reduction in the liver function parameters aminotransferase (AST), alanine aminotransferase (ALT), and alkaline phosphatase (ALP) and total cholesterol (TC), together with histopathology studies, supported diabetic recovery by SD11. A closer examination of two major markers for the insulin pathway, insulin receptor (INSR) and insulin substrate (IRS)-1, revealed that SD11 could exert its glucose control through the upregulation of these molecules, which were almost demolished in nontreated diabetic livers. Additionally, SD11-treated mice exhibited alleviation of oxidative stress enzymes; downregulation of proinflammatory cytokines, including interleukin (IL)-6, IL-1β, tumor necrosis factor (TNF)-α, and interferon (IFN)-γ; and decreased infiltration of macrophages into liver tissue. These findings were concomitant with the preservation of the tight junction proteins occludin and zona occludin (ZO)-1, which in turn lowered the levels of the inflammatory cytokines IL-1β and TNF-α and prevented colon tissue injury to some extent. Notably, the results for the SD11 control mice were identical to those for the normal control mice. Overall, our findings that SD11 delays liver deterioration and reduces colon lesions in diabetic mice provide evidence for the use of SD11 as an effective strategy to improve diabetes-related symptoms.

## Introduction

A link between diabetes and liver disease has been suggested for a very long time, and it is becoming clear that each condition increases the risk for the other as liver function deteriorates and the incidence of diabetes increases [1,2]. Patients with diabetes are found to have a spectrum of liver diseases, ranging from abnormal liver enzymes to nonalcoholic fatty liver disease (NAFD) [1,2], cirrhosis, liver cancer and acute liver failure [3]. Recently, intestinal barrier dysfunction has been reported to contribute to and promote diabetic liver deterioration through direct anatomical and physiological connections between the gut and the hepatic portal vein, which is commonly known as the gut‒liver axis [4]. Compelling evidence suggests that hyperglycemia, oxidative stress, and inflammation are major contributing factors in the vicious cycle of worsening liver disease [3–5]. Thaiss et al. demonstrated that prolonged hyperglycemia is the main catalyst causing impairment of the intestinal barrier and increased intestinal mucosal inflammation, leading to the dissemination of bacteria and their metabolite products into the systemic circulation, which in turn aggravates liver malfunction [6]. Therefore, the search for alternative and effective natural hypoglycemic foods with promising clinical applications and their development into functional foods have gained increasing attention.

*Lacticaseibacillus rhamnosus* SD11 (formerly *Lactobacillus rhamnosus* SD11) was first isolated and characterized from caries-free children [7,8]. Attempts have been made to investigate the appropriate formulas used and to study the efficiency of this probiotic for promoting human oral health and a great number of clinical trial studies have been published [7–11]. Previous clinical trials in human volunteers have revealed its strong inhibitory effect against periodontopathogens, with no evidence of side effects even after long-term administration (milk powder containing SD11 once daily for up to six months) [9–12]. Recently, studies have shown that SD11 has an anti-inflammatory effect by reducing cytokine and human β-defensin-2-4 levels in human oral epithelial cells [13]. These studies ensure its oral care benefits and safety for oral consumption in humans. Additionally, on the basis of the evidence of our previous research, intervention with SD11 for 4 weeks not only alleviated metabolic dysfunction but also modulated subsequent glucotoxicity through relieving the insulin index and inflammation and suppressing pancreatic β-cell apoptosis in STZ-induced diabetic mice [14]. Moreover, many studies have shown that *Lacticaseibacillus* is a common probiotic in the gastrointestinal tract and has been implicated in liver disease along the gut‒liver axis in both animals and human subjects [15,16]. A study by Lee et al. demonstrated that the ingestion of *L*. *acidophilus*, *L*. *fermentum*, and *L*. *plantarum* ameliorates the progression of nonalcoholic steatosis by lowering cholesterol and liver enzymes [17]. Similarly, Yan et al. reported that *L*. *acidophilus* KLDS1.1003 and KLDS1.0901 529 improve intestinal barrier function, suppress inflammatory responses in the liver and colon, and regulate glucose and lipid metabolism in the liver [18].

Hence, in this present work, we aimed to broaden the applications SD11 in STZ-induced diabetic mice in the aspects primarily associated with hyperglycemic phenotype-driven liver and colon damage. We hypothesized that the antidiabetic properties of SD11 and its ability to repair colon lesions might be closely related to the modulation of the insulin signal transduction pathway, oxidative stress, inflammation, and colonic tight junction proteins. Our discovery that SD11 exerts antidiabetic effects and preserves colon lesions to various degrees provides a theoretical foundation for the practical application of SD11 in preventing diabetic-related complications.

## Materials and methods

### Probiotic strains and cultivation

The identification, culture conditions, and preparation of the experimental strain SD11 were described previously [14]. In the present work, the probiotic strain was recovered from storage at -80°C on de Man, Rogosa and Sharpe (MRS) agar plates and inoculated into 50 mL of MRS broth overnight under anaerobic conditions (80% N_2_, 10% H_2_ and 10% CO_2_) at 37°C. The cultures were then added to 450 mL of MRS broth and incubated under anaerobic conditions at 37°C for 48 h. The cells were harvested by centrifugation (3,000 × *g*, 5 min) from the MRS broth and washed 3 times with 0.85% NaCl before being used. The SD11 solution was freshly prepared by resuspension in distilled water and adjusted to a density of 10^9^ colony-forming units (CFUs)/200 μl prior to use [14]. This effective concentration was selected according to previous animal and clinical studies [10,11,14].

### Animal model and probiotic intervention

**Fig 1:**
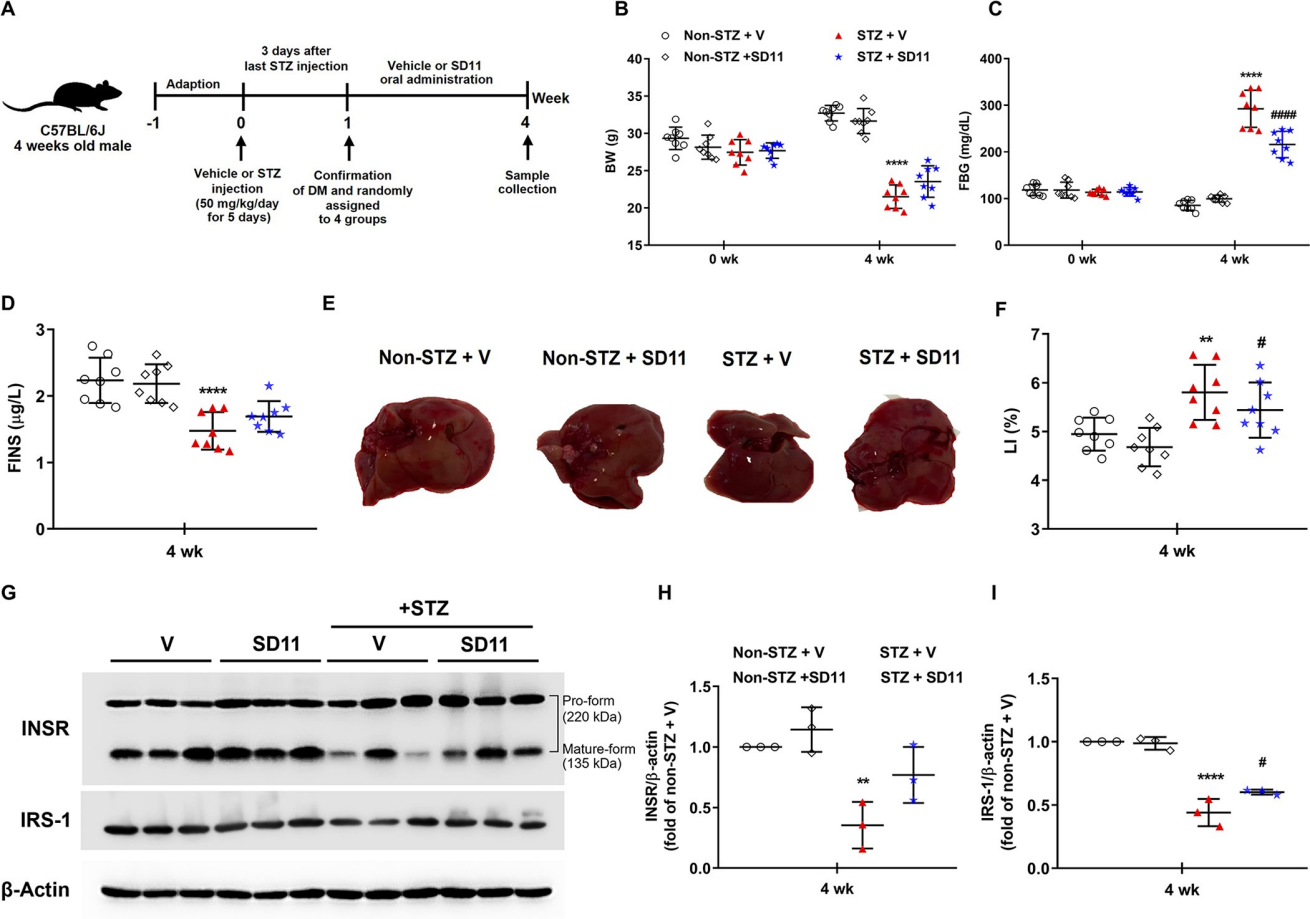
Impact of SD11 on STZ-induced diabetic mice. (A) Experimental design of the diabetic model, (B) body weights (BW), (C) fasting blood glucose levels (FBG), (D) fasting blood glucose levels (FINS), (E) gross specimen of the mouse liver, (F) liver index (LI), and (G) representative images of Western blot analysis of the liver glucose metabolic markers INSR and IRS-1. Densitometry quantification of (H) INSR and (I) IRS-1 corrected by β-actin. The graphs show mean ± S.D. (n = 8 per group for physiological data) or mean ± SEM (n = 3/group, 2 independent experiments for Western blots). ****P < 0.0001, **P < 0.01 vs. the corresponding non-STZ + V group; ^####^*P* < 0.0001, ^#^*P* < 0.05 vs. the corresponding STZ + V group.

In this study, 32 male BALB/c mice (4–5 weeks old) weighing approximately 18–21 g were used. Animal care, experimental design and procedures were approved by the Institutional Animal Care and Use Committee of Prince of Songkla University (MHESI 68014/2307, Ref. 104/2021). Additionally, this animal protocol adheres to standards articulated in the ARRIVE guidelines [19]. The animals were purchased from Nomura Siam International Co., Ltd., (Bangkok, Thailand) and were maintained under standard conditions of temperature (25 ± 2°C) and humidity (50 ± 10%) with alternating 12-h light/dark cycles in the laboratory animal service center of Prince of Songkla University. As illustrated in Fig 1A, all of the animals were acclimatized under laboratory conditions for one week prior to the experiments. Hyperglycemic conditions were induced intraperitoneally for 5 consecutive days with 50 mg/kg STZ (freshly prepared by dissolving in 0.1 M citrate buffer, pH 4.5). The citrate buffer was administered to the control animals in the same manner. Week 0 was defined as the first day of STZ injection. Seventy-two hours after the last STZ injection, the FBG level in the tail vein was measured via a blood glucose meter (Accu-Check Active® and test strips; Roche Diagnostic, Mannheim, Germany). The mice were considered diabetic when their blood glucose levels were above 200 mg/dL [14,20,21] and were randomly assigned to 4 treatment groups of 8 mice as follows (Fig 1A):

In Group 1 (non-STZ + V), normal control mice were fed distilled water (vehicle, V).

In Group 2 (non-STZ + SD11), positive control mice were given 10^9^ CFU/200 μl of SD11.

In Group 3 (STZ + V), diabetic control mice were fed distilled water.

In Group 4 STZ + SD11 diabetic mice were given 10^9^ CFU/200 μl SD11.

Normal and diabetic control mice received equal volumes of distilled water in place of the probiotic SD11 daily. The mice were fed vehicle or SD11 by gavage once daily for 4 weeks. After 4 weeks, they were euthanized with thiopental (70 mg/kg b.wt.). Blood and organs were collected for further experiments.

### Serum and liver biochemistry measurements

Assessment of liver function markers was performed by collecting blood samples from overnight fasted mice and then allowing the blood to clot at room temperature (RT) for 30 min. Serum was collected by centrifugation at 2,000 × *g* at 4°C for 30 min to collect the serum, which was subsequently stored at -80°C until use. The activities of serum AST, ALT, and ALP were estimated using standard methods on a Cobas MIRA automated analyzer (Roche Diagnostics). In addition, serum total cholesterol (TC) was measured following the manufacturer’s instructions (Cell Biolabs, Inc., San Diego, CA, USA) [20].

The antioxidant enzymes (superoxide dismutase (SOD) and catalase (CAT)) and markers of lipid peroxidation production (malondialdehyde (MDA)) were evaluated via commercially available kits from Sigma‒Aldrich (SOD and MDA assay kits) and Cayman Chemicals Ltd. (catalase assay kits) [21]. Briefly, liver tissues were isolated from euthanized mice, rinsed with ice-cold PBS, homogenized in lysis buffer obtained from the kits, and centrifuged at 10,000 × *g* for 15 min at 4°C. Then, the supernatants were further processed following the manufacturer’s instructions [21].

### Histopathological examination

The specimens were immediately removed and fixed in 10% neutral formalin for 24 hr at RT. After fixation, the tissues were dehydrated in ascending grades of alcohol, cleared in xylene and embedded in paraffin blocks. The embedded tissues were sectioned into 5 μm thick sections and stained with hematoxylin and eosin (H&E), periodic acid-Schiff (PAS) and Masson’s trichrome (MT) according to standard histological laboratory procedures. Histopathological changes were observed via light microscopy (Olympus D73 equipped with CellSens software). For evaluation purposes, the nonalcoholic fatty liver disease activity score (NAS) was used to grade the pathological changes among the treatment groups [22]. Steatosis and inflammation were calculated according to the NASH clinical research network scoring system for NAFLD [22,23]. Steatosis was divided into 4 grades (0: <5%, 1: 5%~33%, 2: 34%~66%, and 3: >66% steatosis). Inflammation was classified into 4 stages (0: none, 1: 1~2 foci/×20, 2: 2~4 foci/×20, and 3: >4 foci/×20 fields) [22,23]. Additionally, the intensity of the PAS- and MT-positive areas from 36 fields in total (3 fields per section, 3 sections per mouse, 4 mice per treatment group) was quantified using NIH ImageJ software. Examinations of the sections were conducted in a blinded manner.

### Immunohistochemical assays

Immunohistochemistry was conducted according to the kit instructions (Vector Laboratories, Inc., Burlingame, USA). Antibodies against IL-6, IL-1β, TNF-α, and F4/80^+^ were acquired from Cell Signaling Technology. Briefly, tissue sections were deparaffinized with xylene and rehydrated in an ethanol series. Antigen retrieval was performed by heating the tissues with citrate buffer, pH 6.0, for 20 min, followed by blocking the endogenous peroxidase activity in 0.3% H_2_O_2_ in methanol for 30 min. After three washes with PBS with 0.1% Triton X-100 (PBST) for 5 min each, blocking buffer (Vector Laboratories, Inc.) was applied for 1 h at RT. The samples were incubated with primary antibodies against IL-6, IL-1β, TNF-α, and F4/80^+^ at 4°C overnight in a moist chamber. The slides were then rinsed with PBST and incubated with secondary antibody (Vector Laboratories, Inc.) for 1 h at RT. After being washed with PBST, the tissues were stained with diaminobenzidine (DAB) until the stain developed. The slides were rinsed with distilled water and counterstained with hematoxylin. The sections were then dehydrated in a series of graded ethanol solutions, cleared in xylene and mounted for microscopic examination under an Olympus DP73 microscope equipped with CellSens software. Positive staining was represented by a brown signal and was photographed. A total of 36 fields per experimental group (3 fields per section, 3 sections per mouse, 4 mice per group) were randomly selected, and the mean intensity in each field was recorded using NIH ImageJ software [21].

### Western blots

Additional antibodies for Western blot analysis were obtained against the following proteins: INSR and IRS-1 (Abcam); and IFN-γ, ZO-1, occludin and β-actin (Cell Signaling Technology). The tissues were lysed and homogenized in ice-cold radioimmunoprecipitation assay (RIPA) buffer (Sigma‒Aldrich) supplemented with 1x protease inhibitor cocktail (Merck-Millipore, USA). After centrifugation at 14,000 × *g* for 30 min at 4°C, the supernatants were collected, and total protein was measured using the BCA protein assay kit (Pierce Biotechnology, USA). The same amount of protein (30 μg of liver or 40 μg of colon) was separated via 8–15% SDS‒PAGE and transferred to PVDF membranes (Merck-Millipore). The membranes were blocked with 5% skim milk for 1 h. The blots were incubated with primary antibodies against INSR, IRS-1, IL-6, IL-1β, TNF-α, IFN-γ, F4/80^+^, occludin, ZO-1, and β-actin overnight at 4°C. Then, the membranes were incubated for 1 h with the corresponding HRP-conjugated secondary antibodies (Sigma‒Aldrich). All signals were visualized with Luminata Crescendo Western HRP substrate (Merck-Millipore) according to the manufacturer’s instructions. The blot images and the density of the target bands were analyzed via a gel document system for chemiluminescence (Alliance Q9-Atom, UVITEC Cambridge). The data were normalized with β-actin as the internal control, and the results were calculated as the fold change compared with non-STZ + V (normal control group). Statistical analysis was then performed.

### Statistical analysis

The results are shown as the means ± standard errors (SDs) unless otherwise noted. A t test was used for comparisons between two groups, and multiple group variance analysis was performed via one-way ANOVA followed by Tukey’s multiple comparisons test with GraphPad Prism 9.0 (GraphPad Software, San Diego, CA) and was considered significant at *P* < 0.05.

## Results

### SD11 affects the physiological and metabolic profiles of STZ-induced diabetic mice

The BW, FBG, FINS, and LI (%) were assessed to determine the physiological and metabolic changes in all of the treatment groups at the end of the experiment. As expected, STZ + V mice exhibited clinical symptoms of hyperglycemia, including a significant decrease in BW (Fig 1B), increased FBG (Fig 1C), decreased FINS (Fig 1D), and liver hypertrophy (Fig 1E and 1F), in comparison to non-STZ + V mice. Mice that received SD11 supplementation had significantly improved FBG levels and percentage of LI compared with that of the STZ + V mice (Fig 1B–1E). Although no significant difference was detected, the SD11-treated group showed a slightly greater BW than that of the STZ + V group (Fig 1B).

We then investigated the ability of liver function in modulating glucose metabolism through the expression of proteins involved in the insulin signaling pathway, INSR and IRS-1 (Fig 1G–1I) in liver tissue. Compared with non-STZ-induced mice, STZ + V-induced mice presented significantly lower levels of both markers (Fig 1G–1I). However, the STZ + SD11 group was able to maintain their expression. Specifically, the expression of IRS-1 in the livers of SD11-treated mice was significantly greater than that in the livers of untreated STZ-treated mice (Fig 1G and 1I). These findings indicate that SD11 has potential benefits in blood glucose control at least in part because of the preservation of insulin receptors, thereby enhancing glucose uptake ability.

### SD11 effectively maintains liver function in STZ-induced diabetic mice

The levels of alkaline phosphatase (ALP), alanine aminotransferase (ALT), aspartate aminotransferase (AST), and total cholesterol (TC) in serum are clinically used as indicators of hepatocyte injury [17,21]. As shown in Table 1, compared with normal control mice, STZ + V mice exhibited liver damage, as evidenced by significant increases in the serum levels of ALP, ALT, AST, and TC. However, the activities of ALT and AST, as well as the level of total cholesterol (TC), were significantly lower in the SD11 group than in the STZ + V group. The level of ALP was reduced, although the difference did not reach a significant level. Furthermore, to study the effects of SD11 administration on enzyme activity and free radical production, SOD and CAT activities and MDA levels were measured in liver tissues. At the end of the experiment, the SOD and CAT activities in the STZ**-**induced diabetic mice were significantly lower, whereas the MDA levels were markedly greater than those in the normal control group, indicating that the liver was disrupted by oxidative stress. The levels of SOD and MDA in diabetic livers were significantly improved, whereas the level of CAT was partially restored by SD11 administration. Specifically, the relevant parameters in the positive control group, non-STZ + SD11, were approximately equal to those in the normal control group. These findings demonstrated that SD11 exhibited strong potential antioxidant effects against oxidative stress in the liver, thereby ameliorating hepatic dysfunction.

**Table 1:** Liver function test and antioxidant enzyme levels of mice.

Group	Serum profiles				Liver oxidative enzymes		
	AST (IU/L)	ALT (IU/L)	ALP (IU/L)	TC (mg/dL)	SOD (U/mg)	CAT (U/mg)	MDA (nmol/mg)
Non-STZ + V	108.88 ± 10.34	36.48 ± 4.11	105.42 ± 3.75	96.47 ±13.77	11.37 ± 1.03	2.39 ± 0.36	0.21 ± 0.03
Non-STZ + SD11	103.25 ± 14.22	41.40 ± 4.58	106.13 ± 14.71	91.90 ± 11.71	11.49 ± 0.63	2.50 ± 0.33	0.24 ± 0.04
STZ + V	223.94 ± 54.18****	95.00 ± 15.04****	149.17 ± 11.95****	223.50 ± 25.93****	6.02 ± 0.80****	1.81 ± 0.14**	0.68 ± 0.11****
STZ + SD11	155.88 ± 36.96^##^	71.25 ± 16.59^##^	120.75 ±17.53	154.88 ± 32.26^####^	8.36 ± 1.19^###^	2.15 ± 044	0.33 ± 0.05^####^

AST, aspartate aminotransferase; ALT, alanine aminotransferase, ALP, alkaline phosphatase; TC, total cholesterol; SOD, superoxide dismutase; CAT, catalase, MDA, malondialdehyde.

Data are shown as the means ± SD, n = 8/group.

****P < 0.0001, **P < 0.01 vs. the corresponding to non-STZ + V group.

^####^*P* < 0.0001, ^###^*P* < 0.001, ^##^*P* < 0.01 vs. the corresponding to STZ + V group.

### SD11 delays STZ-induced pathological changes in the liver

**Fig 2:**
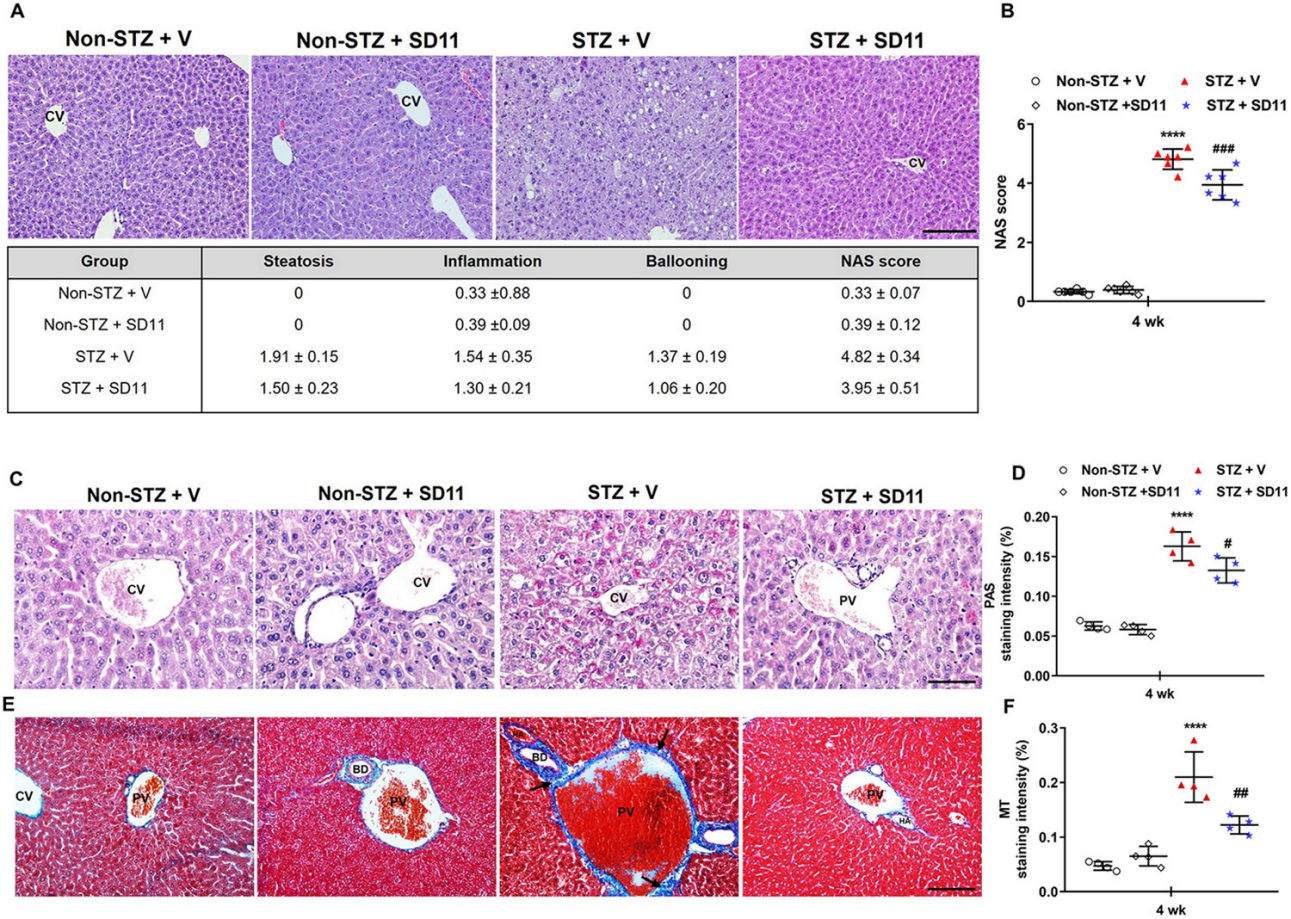
SD11 attenuates the progression of liver deterioration in STZ-treated mice. Representative images and quantification of liver sections subjected to (A-B) H&E, (C-D) PAS, and (E-F) MT staining. H&E and MT images were taken at 200× magnification, whereas PAS images were taken at 400× magnification. Scale bars, 50 μm. Each value represents the mean ± SEM. n = 36 fields (4 mice/group). ****P < 0.0001 vs. the corresponding non-STZ + V group; ^###^*P* < 0.001, ^##^*P* < 0.01, ^#^*P* < 0.05 vs. the corresponding STZ+ V group. CV: Central vein; PV: Portal vein; BD: Bile duct. Black arrows indicate the increased deposition of collagen around the portal areas.

Histopathological changes by H&E were used to assess the degree of liver damage with or without SD11 supplementation (Fig 2A and 2B). In the control group, the size and shape of the hepatocytes were consistent, the hepatic sinusoids were neatly organized, and the hepatic lobule structure was intact, whereas the STZ-induced liver displayed moderate to marked large fat vacuoles that accumulated in hepatocytes and created a ballooning appearance (Fig 2A). These differences in steatosis, ballooning cells, and inflammation scores were significant compared with those of normal control mice (Fig 2B). In contrast, the NAS score in the livers of the SD11-treated mice was significantly lower than that in the livers of the untreated diabetic mice (Fig 2B).

Given that liver glycogenesis is a crucial process in regulating FBG levels, we next investigated the effect of SD11 on glycogen deposition in hepatocytes by PAS staining (Fig 2C and 2D). The livers of normal and positive control mice presented normal glycogen staining scattered in the cytoplasm of hepatocytes, while glycogen was intensely stained in the diabetic liver and was found mainly in the surrounding portal vein (PV) and central vein (CV). Compared with that in the livers of the STZ-treated mice, the distribution of glycogen in the livers of the SD11-treated mice was significantly lower (Fig 2C and 2D). Additionally, MT staining was conducted to determine the amount of collagen produced, which reflects the incidence of liver fibrosis. As shown in Fig 2E (black arrows), diabetic livers presented marked fibrosis, especially at the edge of the portal area, compared with those of control mice. However, the diabetes-induced increase in collagen accumulation was significantly attenuated by SD11 treatment (Fig 2A–2F). In conclusion, the STZ-induced mice presented extremely abnormal liver histology, but SD11 supplementation significantly delayed the deterioration of liver architecture and ameliorated diabetes-induced liver glycogenesis and fibrosis.

### SD11 attenuates inflammatory responses in the livers of STZ-induced diabetic mice

**Fig 3:**
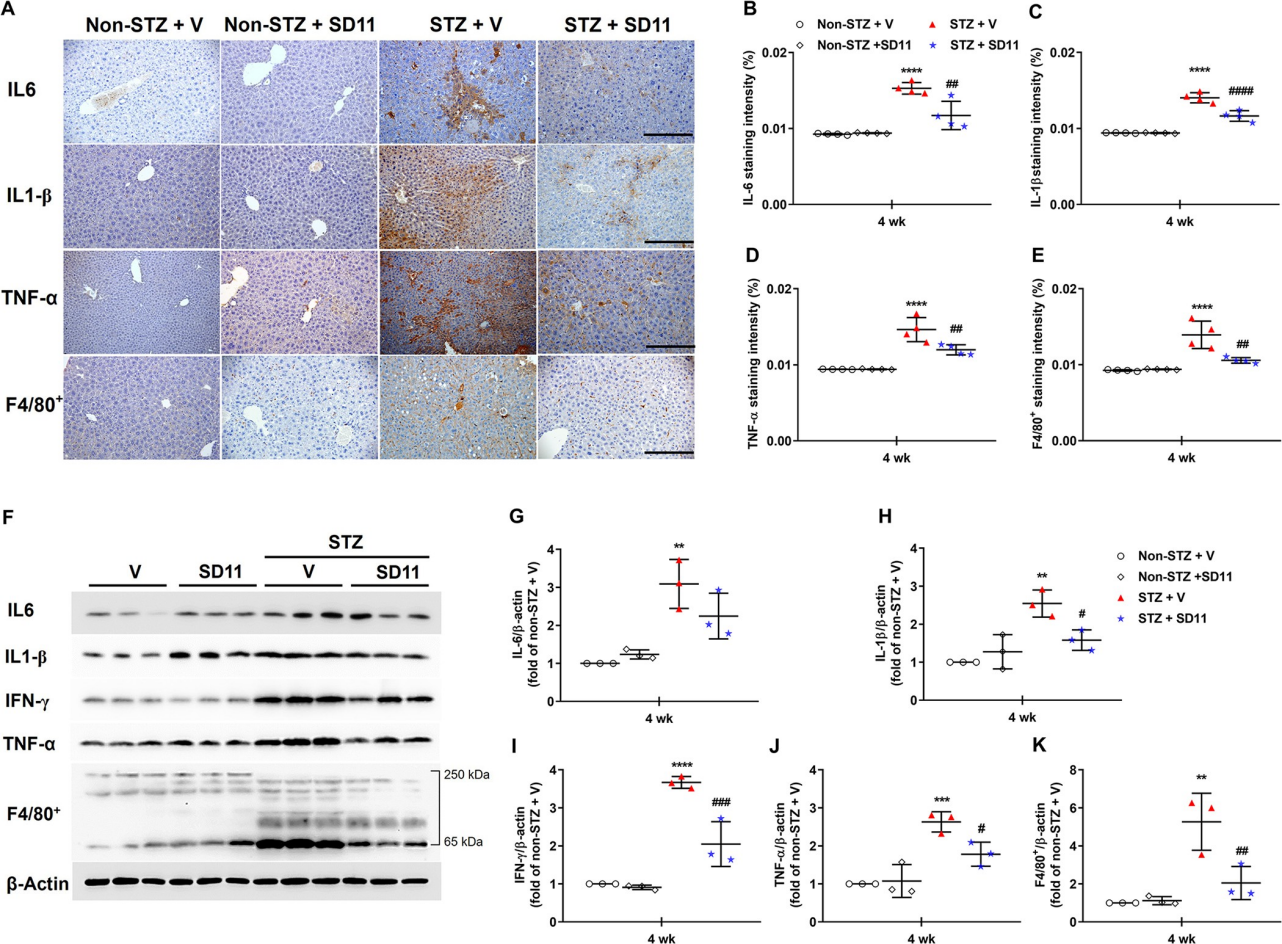
SD11 reduces immunoresponses in the livers of STZ-induced mice. (A) Representative images of liver sections immunostained for IL-6, IL-1β, TNF-α, and the macrophage marker F4/80^+^. Quantification of (B) IL-6, (C) IL-1β (D) TNF-α, and (E) F4/80^+^. All images were taken at 200× magnification. Scale bars, 50 μm. The values represent the mean ± SEM**,** n = 36 fields (4 mice per group). (F) Representative images of Western blot analysis of the cytokines IL-6, IL-1β, TNF-α, IFN-γ and F4/80^+^. Densitometry quantification of (G) IL-6, (H) IL-1β, (I) TNF-α, (J) IFN-γ, and (K) F4/80^+^ corrected by β-actin. The data are presented as the mean ± SEM, n = 3/group, 2 independent experiments. ****P < 0.0001, ***P < 0.001, **P < 0.01 vs. the corresponding non-STZ + V group; ^####^*P* < 0.0001, ^###^*P* < 0.001, ^##^*P* < 0.01, ^#^*P* < 0.05 vs. the corresponding STZ+V group.

Activated macrophages produce pronominally proinflammatory cytokines, resulting in the upregulation of inflammatory reactions under hyperglycemic conditions [24]. There is abundant evidence that certain proinflammatory cytokines, such as IL-6, IL-1β and TNF-α, are involved in the process of liver damage [24,25]. Immunohistochemical staining for IL-6, IL-1β, and TNF-α revealed that the number of positive inflammatory cells was increased after 4 weeks in nontreated STZ-treated mice but was reduced by SD11 (Fig 3A–3D). Importantly, immunohistochemical staining also revealed that the administration of SD11 inhibited the STZ-induced accumulation of F4**/**80^+^ macrophages in the liver (Fig 3A and 3E). These results were in accordance with the results of the Western blot analysis of inflammatory protein expression. As shown in Fig 3F–3K, the protein expression levels of IL-6, IL-1β, TNF-α, and F4**/**80 in the diabetic mice were greater than those in the normal control mice. Following the administration of SD11, these proinflammatory markers were significantly downregulated (Fig 3H–3K), except for IL-6, whose level of reduction did not reach a significant level (Fig 3G).

### SD11 plays a role in modulating the gut‒liver axis via the upregulation of tight junctions in the colon of STZ-induced diabetic mice

**Fig 4:**
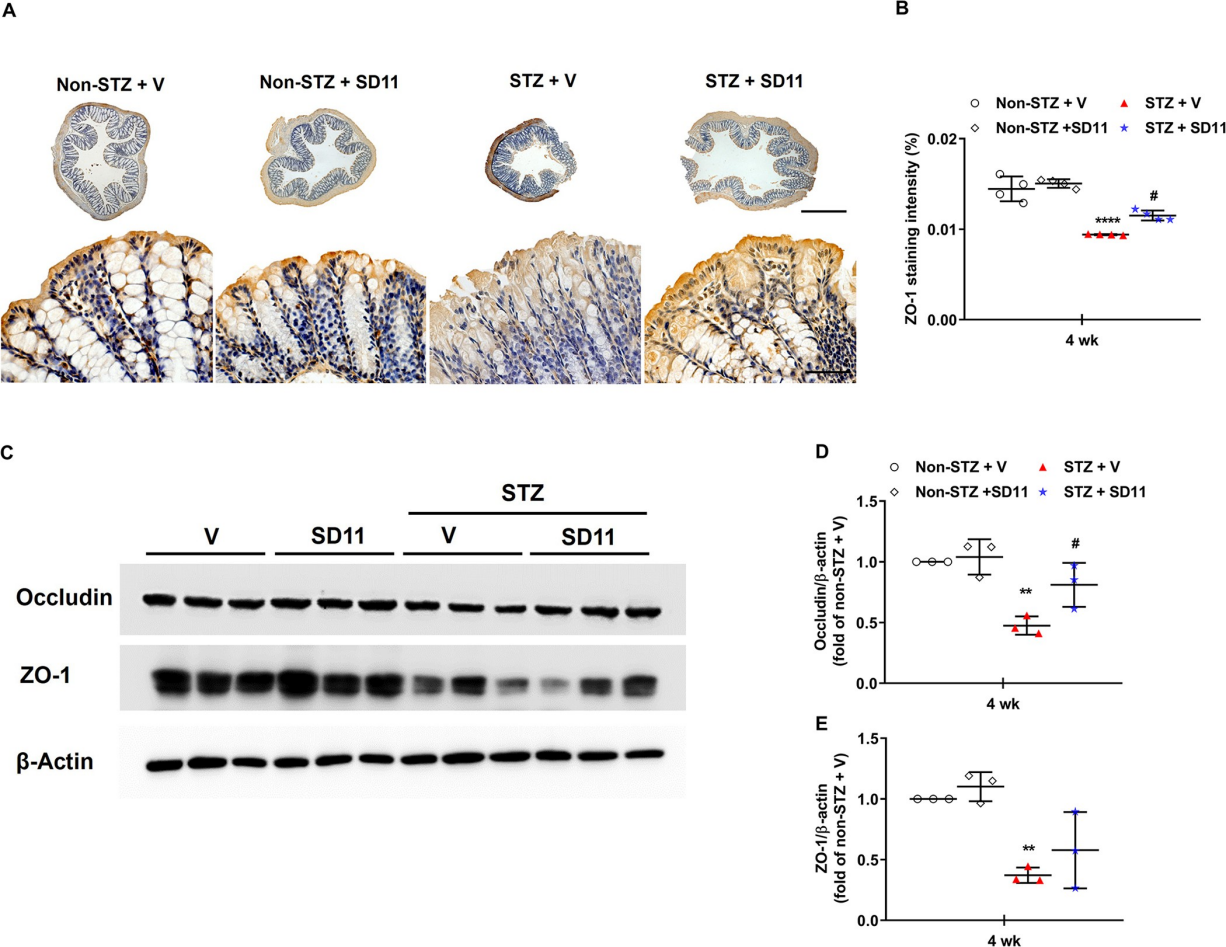
SD11 modulates gut integrity via the upregulation of tight junction proteins in the colon of STZ-induced diabetic mice. (A) Representative images. (B) Quantification of colon sections immunostained for ZO-1. The images were taken at 50× (top panel) and 600× (bottom panel) magnifications. Scale bars, 50 μm. The values are expressed as the mean ± SEM**,** n = 36 fields (4 mice per group). (C) Representative images of Western blot analysis of occludin and ZO-1. Densitometric quantification of (D) occludin and (E) ZO-1 corrected by β-actin. The data are presented as the mean ± SEM, n = 3/group, 2 independent experiments. ****P < 0.0001, **P < 0.01 vs. the corresponding non-STZ + V group; ^#^*P* < 0.05 vs. the corresponding STZ + V group.

Impairment of the epithelial barrier in the colon, which is associated with increased permeability through decreased expression of tight junction proteins, has been implicated as a critical factor that exacerbates inflammation in diabetes [4–6,16]. Occludin and ZO-1 are proteins involved in maintaining the integrity of intact tight junction complexes and barrier function [16]. Hence, we investigated whether SD11 has beneficial effects on the disruption of these tight junction proteins in the diabetic colon. As shown by immunohistochemical staining, ZO-1 was localized within intestinal epithelial cells (Fig 4A and 4B). ZO-1 in normal mice was abundant and widely distributed, whereas it was disrupted and significantly reduced in the colons of diabetic mice (Fig 4A and 4B). Compared with the control, the 4-week intervention with SD11 resulted in significant recovery of epithelial integrity (Fig 4A and 4B). Consistent with the immunohistochemistry results, the protein expression levels of occludin and ZO-1 in colon tissues were lower in the STZ-treated mice than in the normal control mice (Fig 4C–4E). Although only the level of occludin significantly differed, the application of SD11 preserved the loss of expression and altered the distribution of both proteins (Fig 4A and 4B). These results indicated that SD11 could ameliorate the progression of diabetes-related colon lesions.

### SD11 suppresses proinflammatory cytokine production in the colons of STZ-induced diabetic mice

**Fig 5:**
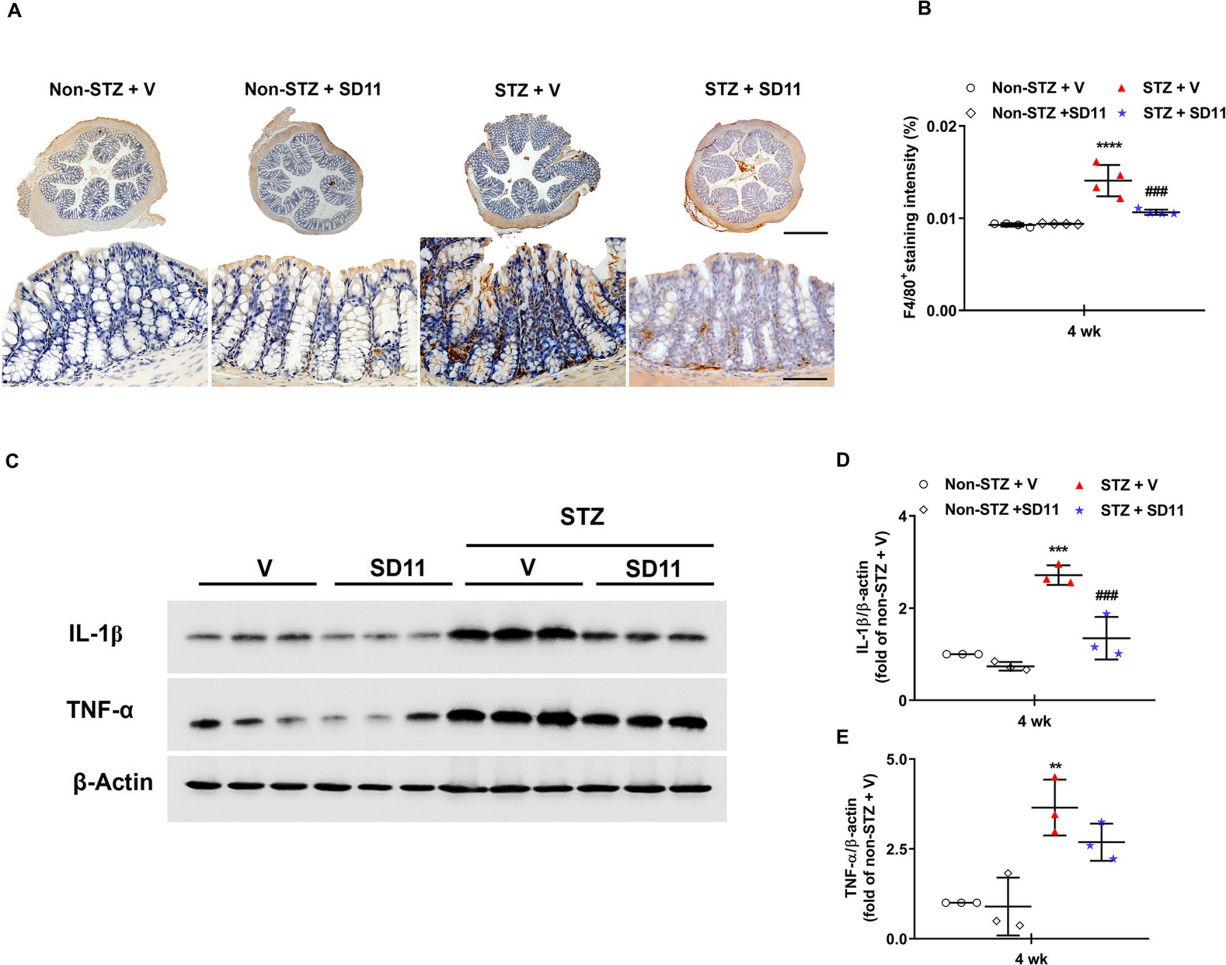
SD11 downregulates colonic inflammatory responses in STZ-induced diabetic mice. (A) Representative images and (B) quantification of colon sections immunostained for the macrophage marker F4/80^+^. The images were taken at 50× (top panel) and 600× (bottom panel) magnifications. Scale bars, 50 μm. The values represent the mean ± SEM**,** n = 36 fields (4 mice per group). (C) Representative images of Western blot analysis of the cytokines IL-1β and TNF-α. Densitometric quantification of (D) IL-6 and (E) TNF-α corrected by β-actin. The data are presented as the mean ± SEM, n = 3 per group. ****P < 0.0001, ***P < 0.001, **P < 0.01 vs. the corresponding non-STZ + V group; ^###^*P* < 0.001 vs. the corresponding STZ + V group.

Since proinflammatory cytokines constitute a positive feedback loop that induces the infiltration of macrophages into tissue [24], the effect of SD11 on the accumulation of macrophages in colon tissues was examined with an F4**/**80^+^ antibody (Fig 5A and 5B). Fig 5A demonstrates that F4**/**80^+^-expressing cells were juxtaposed within the crypt. Compared with that in the normal liver, the number of macrophages in the STZ-induced colon was significantly greater. Moreover, the villi of the colon tissues in the diabetic group were arranged in a disorderly manner and were easy to break, resulting in many vacuolar changes and damaged crypts. Following SD11 administration, there was a much lower abundance of F4**/**80^+^-expressing cells along the colonic crypts, and the villi appeared to be more organized. Furthermore, detection of IL-1β and TNF-α by Western blotting revealed that the expression of proinflammatory cytokines in the proximal colon of the model animals was semi-ideal (Fig 5C–5E). Prolonged hyperglycemic conditions significantly increased the expression levels of all key molecules evaluated. These changes were significantly mitigated by SD11 administration for 4 weeks (Fig 5C–5E). These results further support our hypothesis that SD11 could mitigate the immune response not only in the liver but also in the colon in STZ-induced diabetic mice, thereby alleviating diabetic complications.

## Discussion

The liver is one of the main targets of insulin and controls metabolic profiles; hence, its function plays an important role in the development of metabolic disorders such as obesity and diabetes [1,2]. Extensive studies have demonstrated that chronic hyperglycemia is one of the major driving forces of physiological alterations, metabolic distortion, and liver dysfunction [2]. Recently, in the research field of diabetes and the gut microbiome, the term gut‒liver axis has attracted considerable scientific interest because of the direct anatomical and physical connection of the liver with the gut through the hepatic portal vein [4]. In particular, evidence has shown that hyperglycemia and insulin resistance disturb the gut‒liver axis and are closely associated with the progression of liver disease [5,6]. Hence, many studies have focused on the potential of various probiotics to neutralize gut‒liver homeostasis as a promising approach for modulating glucose metabolism, inflammation, and cellular damage, thereby ameliorating diabetes-related symptoms. On the basis of this bidirectional communication, the results from different research groups have indicated that some probiotics, such as *B*. *lactis*, *L*. *casei*, *B*. *bifidum*, and *L*. *acidophilus*, have protective effects on the liver and intestine in both clinical and preclinical studies [18,26–28]. Among these strains, *Lactobacillus* has been shown to be the most studied. Nonetheless, the comprehensive mechanism remains elusive. In this context, we investigated the effects and associated underlying mechanisms of SD11 on diabetes-related liver damage and gut integrity.

The probiotic SD11 was first isolated from the human oral cavity more than a decade ago and has been intensively tested for its benefit to human health and for use in dental prophylaxis [7,10–13]. Our previous research revealed that SD11, as well as a probiotic strain of SD1, has the potential to relieve diabetic phenotypes, including glycemic control and systemic inflammatory status, in STZ-induced pancreatic damage in mice [14]. In the present work, we focused on the effects of SD11 on liver health and functional status and its underlying mechanisms in association with changes in the colon. Our analysis of the metabolic performance of diabetic mice revealed that DM was associated with decreased BW, increased FBG, liver hypertrophy, and decreased secretion of FINS. These metabolic abnormalities were reversed with SD11 supplementation. We postulate that significant weight loss in diabetic mice is due to a severe insulin deficiency (Fig 1C and 1D) which reduces all anabolic processes and accelerates fat and protein catabolism [18,27]. A prolonged period of chronic hyperglycemia and resulting oxidative stress is also attributed to an increased degradation of muscle proteins in diabetic mice [14,20]. The slight improvement in BW by probiotic SD11 could be due to better glycemic control in these mice (Fig 1), which led to improvement in their energy intake and metabolic energy expenditure; hence, compensating for the loss in fat and muscle mass.

The role of SD11 in the body may be multifaceted and multitargeted; hence, the hypoglycemic mechanism of SD11 has been preliminarily investigated from the perspective of the insulin signaling pathway. The key signaling transductors INSR and IRS-1 were analyzed in the liver to further assess glucose uptake status in our experimental STZ mouse model. IRS-1 is a downstream protein of INSR that acts as a cellular adaptor molecule to mediate metabolic actions, such as glucose uptake, lipid metabolism and cell proliferation, after INSR activation [29]. Studies have shown that both markers may inhibit hyperglycemia and hyperinsulinemia while promoting endogenous glucose production and are crucial, especially for reducing postprandial blood glucose concentrations [29,30]. Our results suggested that SD11 could upregulate the protein expression levels of INSR and IRS-1 in liver tissues compared with those in untreated animals (Fig 1). Collectively, our mouse model successfully recapitulates the characteristics of diabetes-related profiles, which are consistent with those previously reported in the literature and further support that the upregulation of INSR and IRS-1 is related to the glucose-lowering effect of the probiotic SD11.

Elevated AST, ALT, ALP, and TC levels in the serum are widely established as biochemical markers that reflect hepatic injury, with possible involvement in the progression of liver disease [17]. In the present study, compared with normal mice, STZ-treated mice presented elevated levels of all biochemical markers for liver function, which is consistent with the findings of the Jiang research group [27]. However, these biochemical markers were recovered in the SD11 group. Consistent with these findings, pathologic analysis revealed a significant increase in the NAS score in diabetic livers, whereas SD11 ameliorated diabetes-induced steatosis and lobular inflammation (Fig 2).

Glycogenolysis and glycogen synthesis are controlled by complex mechanisms. The orchestration of the inhibited glycogenolytic molecules and activated glycogen synthesis molecules determines the net glycogen accumulation in the liver. Generally, glucose is the main inhibitor of hepatic glycogenolysis, and insulin is the main activator of hepatic glycogen synthesis [18,30]. Under DM conditions, blood glucose levels are high, causing increased flux of glucose into hepatocytes, which leads to increased glycogen production and excessive glycogen storage in the liver [18]. Fig 2 shows that the glycogen content in diabetic model mice was greater than that in normal mice. Nevertheless, the level of glycogen in the mice in the SD11 group was significantly lower than that in the model group, and the results were identical to the changes in the NAS score and serum biochemical markers. These findings revealed that SD11 restored the homeostasis of hepatic glycogen production to nearly normal levels, which may also be one of the mechanisms related to its hypoglycemic effect, and that SD11 provides an effective means to reduce liver steatosis in diabetic mice.

The accumulation of lipid droplets in the cytoplasm of hepatocytes observed in our mouse model resembles the pathology of NAFLD. This result indicates the sequence of initially impaired metabolic functions for facilitating fat storage in hepatocytes, which causes constant hepatocytic injuries, increasing oxidative stress and the activation of inflammatory cascades, working in a positive feedback fashion [1,18,30]. Specifically, liver-resident macrophages, Kupffer cells, play critical roles in determining the balance between the mechanisms of progression and resolution of tissue injury [23–25]. Once oxidative stress occurs, it activates Kupffer cells and tissue injury by activating various cytokines, including IL-6, IL-1β, and TNF-α [24]. In a previous clinical study, probiotic treatment with *Lactobacillus*, *Lactococcus*, *Propionibacterium*, and *Bifidobacterium* was shown to be associated with reduced levels of inflammatory cytokines such as IL-1β, TNF-α, IL-6 and IL-8 in patients with NAFLD [24,31,32]. In our present work, oxidative stress was assessed by measuring key antioxidant enzymes in liver tissues (Table 1). A decrease in SOD and CAT, with increased MDA levels in the livers of the STZ-induced mice, was observed. Following SD11 supplementation, the levels of oxidative markers produced by STZ were significantly ameliorated. Moreover, elevated levels of macrophages (F4/80^+^) and proinflammatory cytokines, including TNF-α, IL-1β, and IL-6, in liver tissues were observed by both immunohistochemical staining and Western blotting in diabetic livers compared with those in normal control livers (Fig 3). Within this context, SD11 partially alleviated this immune response in the liver. The reduction in these proinflammatory cytokines after intervention with SD11 for 4 weeks was associated with the amount of oxidative stress markers, which demonstrated that the suppression of inflammatory responses played an essential role in delaying liver deterioration.

It is conceivable that gut‒liver communication plays an important role in the pathophysiology of diabetes-induced liver damage [17,18]. The intestinal barrier is essential for absorbing nutrients necessary for humans and preventing the invasion of microorganisms into the lumen [18]. Alterations in intestinal barrier function are associated with increased intestinal permeability, which plays a crucial role in the initiation and progression of hepatic and extrahepatic damage [30]. Destruction of junctional proteins induces altered barrier integrity and intestinal inflammation, leading to the induction of NAFLD. Xin *et al*. reported that the level of TJ protein expression in IECs was related to the occurrence and development of NAFLD [33]. Specifically, in a group of 92 patients, ZO-1 and occludin expression was decreased in NAFLD patients [33]. Hence, in this study, we investigated the effects of SD11 on the mechanical and immunological barriers in the diabetic colon. We found that the villi of colon tissues were disordered and easy to break, and many vacuolar changes and damaged crypts were observed in diabetic mice (Fig 4). This phenomenon might be strongly associated with the loss of the tight junction proteins occludin and ZO-1, both of which are key for maintaining intestinal barrier integrity, as demonstrated in Fig 4. A significant increase in immune cells (macrophages) and proinflammatory cytokines in the non-treated diabetic colon was also detected (Fig 5). However, a 4-week intervention with SD11 upregulated occludin and ZO-1 expression and mitigated immune responses in the colon (Fig 5). The above results suggest that probiotics effectively improve outcomes through modulating epithelial integrity and inflammatory pathways in the colon tissue of diabetic mice.

Our current study has some limitations as follows: We did not investigate the effects of SD11 on the gut microbiota profile. It has been suggested that microbiota dysbiosis contributes to the onset and maintenance of the insulin pathway and glycemic control. These changes in the diversity and abundance of the microbiota possibly affect the production of lipopolysaccharide (LPS), which can initiate endotoxin inside the intestinal lumen [1–3]. Specifically, studies have reported that diabetic patients have high levels of *Enterobacteriaceae*, *Enterococcus*, and *Bacteroides* whereas the levels of *Bifidobacterium* and *Pseudomonas* are decreased [5,6,27,31]. To overcome this limitation, future studies are needed to determine whether SD11 could restore the microbial compositional changes in STZ-induced diabetes. Furthermore, clinical data show that dysbiosis or microbe**-**associated bacterial metabolites are harmful to the liver and contribute to the progression of liver steatosis and fibrosis as well as the inhibition of the expression of TJ proteins and increased intestinal permeability via toll**-**like receptors in the small intestine [4]. Hence, it would be attractive to investigate the inhibitory effect of SD11 on the cellular redistribution of occludin-ZO-1 in association with toll-like receptor complexes in other parts of the intestine with additional molecular markers. This set of data will enhance our understanding and significant insights into the effectiveness of SD11 in managing diabetes-related symptoms. Investigating the effects of orally administered SD11 on genetic or sole diet**-**induced diabetes models to clarify whether SD11 simply alleviated the oxidative stress associated with STZ intoxication would also be interesting.

## Conclusion

First, our current study recapitulated the potential of probiotic**-**based therapy for diabetes-induced liver and colon damage. Compared with the untreated diabetic group, SD11 supplementation significantly improved liver function as indicated by the NAS, serum biochemistry markers, and insulin signaling pathway. The improvement was coupled with the recovery of the histological signature and phenotypes. This preventive effect remedies the activation of oxidative stress and the immune response in the liver. The normalized function was also accompanied by the restoration of tight junction proteins and the release of proinflammatory cytokine signals, which are implicated in gut microenvironmental homeostasis and might be potential mechanisms through which SD11 improves diabetes-related symptoms.

## Supporting information

S1 File Supplemental raw data. Unprocessed raw data used in the manuscript.
(XLSX)

S1 Fig Raw images. Original unedited blot results.
(PDF)
