# Supplementary material for: The probiotic Lacticaseibacillus rhamnosus SD11 alleviates the progression of liver and colon damage through modulation of inflammation and tight junction proteins in streptozotocin-induced diabetic mice
Source: PLoS One. 2024 Nov 21;19(11):e0313395. doi: 10.1371/journal.pone.0313395 (PMC11581286; doi:10.1371/journal.pone.0313395)

S2 Fig. Unedited images of Fig 1. M: Protein marker.

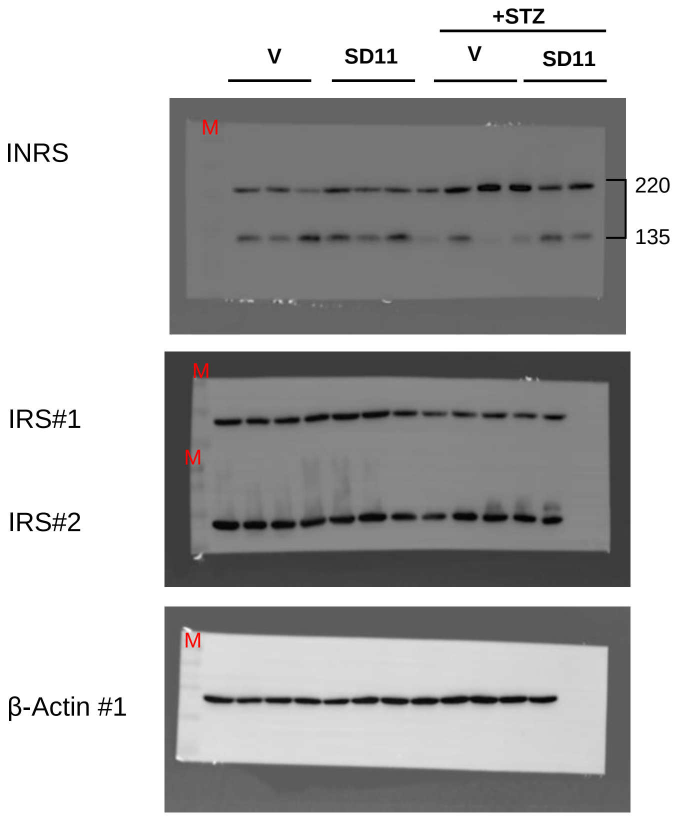

S2 Fig. Unedited images of Fig 3. M: Protein marker.

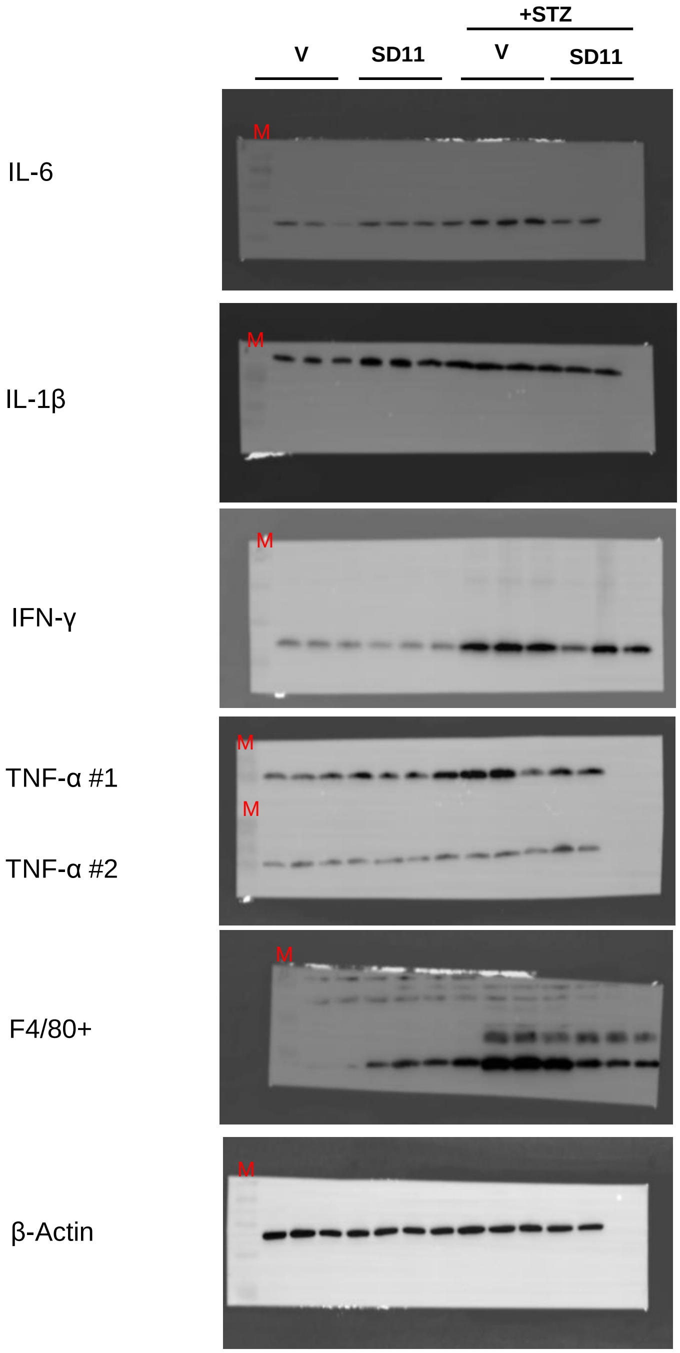

S2 Fig. Unedited images of Fig 4 and Fig 5. M: Protein marker.

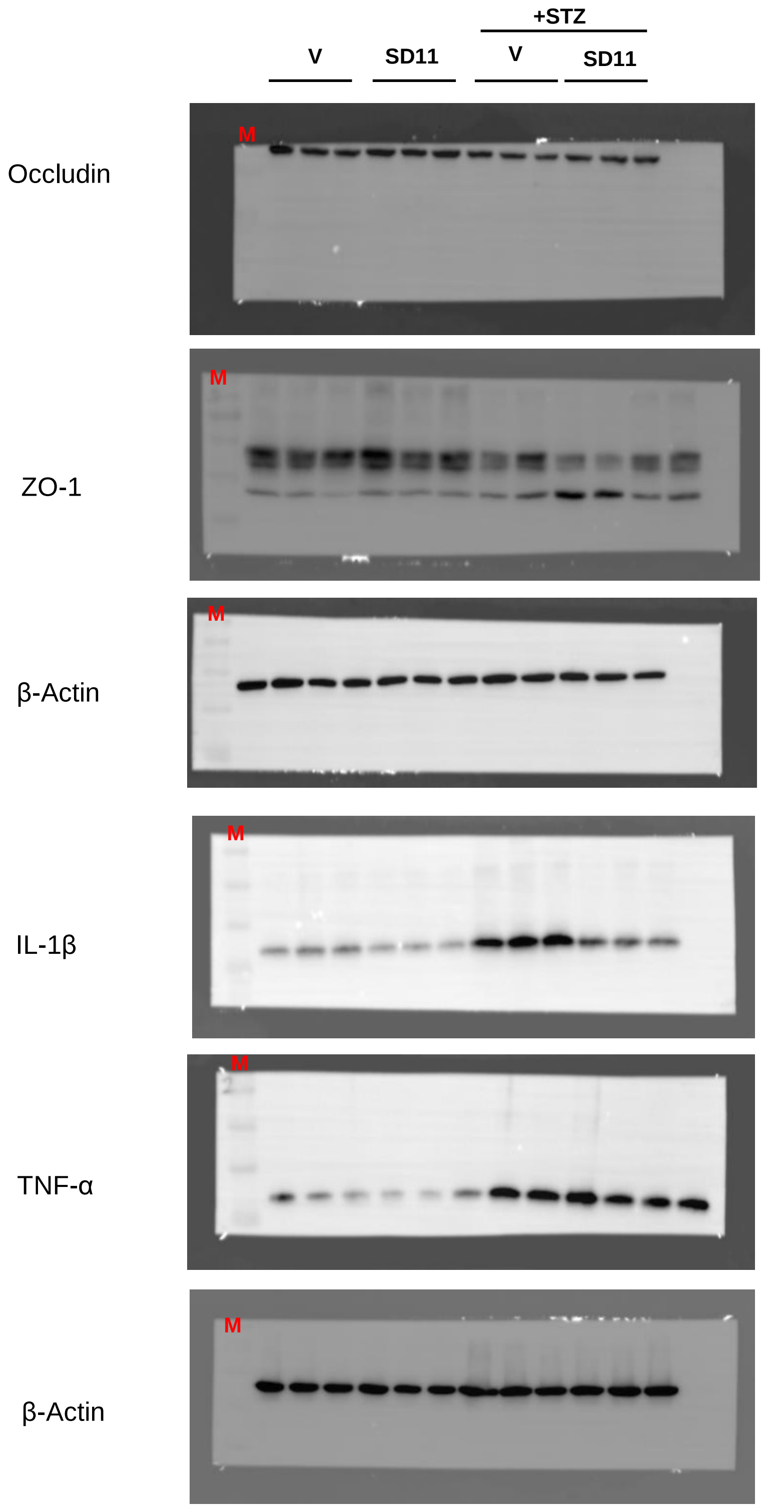

Supplement: S1 Fig — Original unedited blot results. (PDF) [file pone.0313395.s002.pdf]
